# Intrauterine Growth Retarded Progeny of Pregnant Sows Fed High Protein:Low Carbohydrate Diet Is Related to Metabolic Energy Deficit

**DOI:** 10.1371/journal.pone.0031390

**Published:** 2012-02-06

**Authors:** Cornelia C. Metges, Iris S. Lang, Ulf Hennig, Klaus-Peter Brüssow, Ellen Kanitz, Margret Tuchscherer, Falk Schneider, Joachim M. Weitzel, Anika Steinhoff-Ooster, Helga Sauerwein, Olaf Bellmann, Gerd Nürnberg, Charlotte Rehfeldt, Winfried Otten

**Affiliations:** 1 Research Unit Nutritional Physiology “Oskar Kellner”, Leibniz Institute for Farm Animal Biology (FBN), Dummerstorf, Germany; 2 Research Unit Reproductive Biology, Leibniz Institute for Farm Animal Biology (FBN), Dummerstorf, Germany; 3 Research Unit Behavioural Physiology, Leibniz Institute for Farm Animal Biology (FBN), Dummerstorf, Germany; 4 Institute of Animal Science, Physiology & Hygiene Unit, Rheinische Friedrich-Wilhelms-Universität, Bonn, Germany; 5 Leibniz Institute for Farm Animal Biology (FBN), Dummerstorf, Germany; 6 Research Unit Genetics and Biometry, Leibniz Institute for Farm Animal Biology (FBN), Dummerstorf, Germany; 7 Research Unit Muscle Biology & Growth, Leibniz Institute for Farm Animal Biology (FBN), Dummerstorf, Germany; Paris Institute of Technology for Life, Food and Environmental Sciences, France

## Abstract

High and low protein diets fed to pregnant adolescent sows led to intrauterine growth retardation (IUGR). To explore underlying mechanisms, sow plasma metabolite and hormone concentrations were analyzed during different pregnancy stages and correlated with litter weight (LW) at birth, sow body weight and back fat thickness. Sows were fed diets with low (6.5%, LP), adequate (12.1%, AP), and high (30%, HP) protein levels, made isoenergetic by adjusted carbohydrate content. At −5, 24, 66, and 108 days post coitum (dpc) fasted blood was collected. At 92 dpc, diurnal metabolic profiles were determined. Fasted serum urea and plasma glucagon were higher due to the HP diet. High density lipoprotein cholesterol (HDLC), %HDLC and cortisol were reduced in HP compared with AP sows. Lowest concentrations were observed for serum urea and protein, plasma insulin-like growth factor-I, low density lipoprotein cholesterol, and progesterone in LP compared with AP and HP sows. Fasted plasma glucose, insulin and leptin concentrations were unchanged. Diurnal metabolic profiles showed lower glucose in HP sows whereas non-esterified fatty acids (NEFA) concentrations were higher in HP compared with AP and LP sows. In HP and LP sows, urea concentrations were 300% and 60% of AP sows, respectively. Plasma total cholesterol was higher in LP than in AP and HP sows. In AP sows, LW correlated positively with insulin and insulin/glucose and negatively with glucagon/insulin at 66 dpc, whereas in HP sows LW associated positively with NEFA. In conclusion, IUGR in sows fed high protein∶low carbohydrate diet was probably due to glucose and energy deficit whereas in sows with low protein∶high carbohydrate diet it was possibly a response to a deficit of indispensable amino acids which impaired lipoprotein metabolism and favored maternal lipid disposal.

## Introduction

The superimposition of pregnancy on the increased nutritional requirements for the maintenance of maternal growth together with fetal growth can lead to maternal-fetal competition for nutrients as mentioned previously [1], [2]. In addition, in immature gravid dams over- and undernourishment (i.e. food oversupply and food restriction) predisposes the still growing adolescent individuals to adverse pregnancy outcome [3]. We have recently developed a model of intrauterine growth restriction (IUGR) by modulating the dietary protein∶ carbohydrate ratio, i.e. high protein and low protein∶carbohydrate ratios, in adolescent pregnant sows [4], [5]. These diets cause reduced maternal body weight (BW) gain, and an aberrant development of body fatness [4]. The pig is increasingly recognized as a biomedical model for energy metabolism and obesity in humans [6]. The maternal low protein model has been used to study effects of a poor nutritional environment *in utero* describing a nutritional situation widespread in the developing world [7], [8]. In contrast, in affluent societies a high protein∶low carbohydrate ratio is popular because it helps to control body weight and fat although long-term effects are not well described [9], [10].

Pregnancy results in partitioning of nutrients to protect the developing fetus. The response of the conceptus to maternal food intake is mediated by the intrauterine environment of nutrients, hormones and growth factors. To explore the underlying mechanisms leading to IUGR in response to diets with imbalanced high or low protein∶carbohydrate ratios in our porcine model, it is necessary to examine the maternal metabolic response during pregnancy. Therefore, the objective of this study in adolescent sows was 1) to monitor diet-dependent changes of maternal plasma metabolite and hormone concentrations during early, mid and late stages of pregnancy, and 2) to test the hypothesis that diet-dependent changes in maternal body weight, body fat, or plasma metabolites and hormones relate to IUGR and provide clues on the underlying metabolic reasons.

## Materials and Methods

### Ethics statement

Procedures performed in this study were in strict accordance with the German animal protection law and approved by the relevant authorities (Landesamt für Landwirtschaft, Lebensmittelsicherheit und Fischerei, Mecklenburg-Vorpommern, Germany; LVL M-V/TSD/7221.3-1.1-006/04; LALLF M-V/TSD/7221.3-1.2-05/06; LALLF M-V/TSD/7221.3-1.2-013/06). All surgery was performed under anesthesia (see below), and all efforts were made to minimize suffering.

### Animals and treatments

Nulliparous German Landrace sows bred at the institute's pig breeding facility were used (EXP1). Sows were estrus synchronized as recently described [4]. At insemination, sows had a mean (± SD) age of 241±4 d, a mean BW of 150±10 kg, and a mean back fat thickness (BF) of 19.9±2.9 mm. Sows still gain BW until the 5^th^ parity, increasing their initial BW at first mating by approximately 60 kg to reach their full mature weight [11]. At the day of first insemination, sows were randomly allocated to three dietary groups. Diets were formulated to be isoenergetic with target crude protein levels of 60 (low protein, LP), 120 (adequate protein, AP), and 300 g/kg (high protein, HP) [4]. The experiment was conducted over 8 temporally successive replicates with at least 9 sows each (3 sows per diet and replicate), and the aim to compare all diets in each replicate. Corn-barley, soybean meal diets (∼13.7 MJ ME/kg) were used. The LP and the HP diets were supplemented by crystalline L-amino acids (AA) in order to achieve AA patterns similar to the AP diet. In LP, AP, and HP diets, the protein∶carbohydrate ratio was 1∶10.4, 1∶5, and 1∶1.3, respectively, and mean amounts of protein intake were 160 g/d, 328 g/d and 768 g/d in groups LP, AP, and HP, respectively. Diets were fed at 2.3 to 2.9 kg/d to achieve an average target energy intake of ∼34 MJ ME/d during pregnancy following recommendations for primiparous sows [12]. The sows were fed twice daily at 07:00 and 15:00 h with 50% of the daily allowance each time, and water was provided ad libitum.

In EXP1, a total of 95 sows were randomly allocated to the three dietary groups. Finally, 19, 25, and 22 sows (n = 66) fed the LP, AP, and HP diets, respectively, produced a litter [4]. The day of the second insemination was defined as day 1 of gestation (1 d post coitum, 1 dpc). Pregnancy was confirmed by ultrasound detection at 28 and 50 dpc and pregnant sows were moved to group pens (2.4 m×6.9 m) with concrete floor and a maximum of 4 sows per group. At 109 dpc, sows were moved to individual farrowing pens and parturition was induced to standardize gestation length and to allow for subsequent fostering of piglets by foster dams [4]. Sows were weighed at insemination and at 2-week intervals during pregnancy until 109 dpc, and BF was measured concurrently by ultrasound which is considered a good measure of body fatness [4], [13]. Reproductive data, dietary details, and sow cumulative BW and BF gain are presented in **Table 1**, and were reported previously [4]. A second experiment (EXP2) was conducted over 5 temporally successive replicates, again with the comparison of all three diets in each replicate (2 sows per diet and replicate). Dietary regimen and sow management were the same as described for EXP1. In EXP2, again a group of nulliparous sows was inseminated and 9 sows per dietary group (LP, AP, HP) were investigated for a diurnal blood metabolite profile at 92 dpc. This time point was selected because in the pig 75% of fetal weight accretion occurs in the last trimester of pregnancy (74–115 dpc) [14], and the demand on maternal metabolism is largest during this period. Thus, we considered the maternal physiological response to the different diets in regard to IUGR observed to be most important during this period.

**Table 1:** Litter size and litter weight at birth, individual birth weight of progeny, and cumulative body weight (BW) and back fat (BF) gain from insemination until 109 dpc of sows fed isoenergetic diets with low protein∶high carbohydrate (6.5% crude protein, LP), adequate protein∶carbohydrate (12.1% crude protein, AP), and high protein∶low carbohydrate (30% crude protein, HP) ratios throughout pregnancy (EXP1) (modified after [4]).

	Diet^1^						*P* value^2^
	LP	SE	AP	SE	HP	SE	D
Litter size	12.6	0.6	11.6	0.6	11.1	0.7	0.283
Litter weight, kg	14.67	0.63	15.96	0.59	13.77	0.71	0.066
Birth weight, kg	1.19^a^	0.04	1.41^b^	0.04	1.21^a^	0.04	<0.001
BW gain, kg	42.1^a^	1.2	68.3^b^	1.2	63.1^c^	1.5	0.049
BF gain, mm	5.1^a^	0.4	5.0^a^	0.4	3.8^b^	0.4	0.050

a–c Within a row, values not sharing a common superscript differ significantly (*P*<0.05) according to Tukey post hoc test.

1 Values are least squares means ± SE per diet over 7 replicates.

2 ANOVA PROC GLM, D = diet. Effect of replicate and interaction of diet×replicate were not significant.

### Blood sampling

After overnight food withdrawal in sows of EXP1, blood samples were taken at −5, 24, 66, and 108 dpc by venipuncture. In EXP2, sows were surgically fitted with an indwelling jugular vein catheter on 84 dpc to allow diurnal blood sampling on 92 dpc. General anesthesia of sows was achieved by an i.v. administration of a combination of ketamine (0.15 ml/kg BW Ursotamin, Serum-Werk Bernburg AG, Bernburg, Germany) and azaperone (0.05 ml/kg BW Stresnil, Janssen-Cilag GmbH, Neuss, Germany). Implantation of the jugular vein catheter was adapted to the method described by Rodriguez and Kunavongkrit [15]. Briefly, medical grade silicon tubing (ID 1.6 mm, OD 3.2 mm, length 850 mm; AMT Düsseldorf, Germany) was inserted under aseptic conditions via a small incision into the jugular vein, fixed with ligatures and tissue glue (Histoacryl, B. Braun AG, Melsungen, Germany) and exteriorized via a small incision on the animal's neck. The external end of the catheter was connected to a two-way stopcock (Angiomed, Karlsruhe, Germany) and placed in a small cotton bag. Antibiotics were administered at surgery and two days thereafter (0.1 ml/kg BW Trimethosel, Selectavet, Weyarn-Holzolling, Germany). Catheters were flushed once daily with a sodium citrate solution. At 92 dpc, diurnal blood collection from the catheter started at 08:00 h and was continued at 2-hourly intervals until the next morning 08:00 h. On the sampling day, a meal with 50% of the daily allowance (2.7 kg/d) was fed at 07:00 h, one hour before the first blood sample, and the second meal was consumed at 13:00 h.

Blood was immediately put on ice and centrifuged at 1500× *g* at 4°C for 20 min and the supernatant was stored at −20°C. Tubes containing F-EDTA (S-Monovette, 1.2 g EDTA/l blood, 1 g fluoride/l blood; Sarstedt AG & Co, Nümbrecht, Germany) were used for the analysis of glucose, triacylglycerol (TG), non-esterified fatty acids (NEFA), total cholesterol (C), low density lipoprotein cholesterol (LDLC), high density lipoprotein cholesterol (HDLC), and insulin-like growth factor-I (IGF-I) concentrations. Serum monovettes (Monovette Z, Sarstedt AG & Co, Nümbrecht, Germany) were used for the measurement of urea and serum protein levels, and tubes containing Li-heparin (14–15 kIU/l blood) were used to determine insulin, glucagon, leptin, cortisol and progesterone (P4) concentrations.

### Metabolite and hormone analyses

Plasma metabolites (glucose, urea, TG, NEFA, C) were analyzed by the Klinik für Rinder (Stiftung Tierärztliche Hochschule Hannover, Germany) using the following kits: glucose (no. 553-230) from MTL Diagnostics (Idstein, Germany), NEFA (no. 434-91795) from Wako Chemicals GmbH (Neuss, Germany), TG (GPO-PAP, no. LT-TR 0015), urea (no. LT-UR 0050), and C (CHOD-PAP, no. LT-GL 01039) from Labor+Technik Lehmann (Berlin, Germany). Analyses were performed automatically by spectrophotometry (Pentra 400, Axon Lab, Reichenbach, Germany). Plasma LDLC (no. 969706) and HDLC (no. 650207) concentrations were measured at the Institut für Klinische Chemie und Laboratoriumsmedizin, University of Rostock, using commercial kits (Beckman Coulter GmbH, Krefeld, Germany). The %HDLC was calculated as the proportion of plasma HDLC in % of total C concentration.

Plasma insulin (no. PI-12K) and glucagon (no. GL-32K) concentrations were measured by RIA using commercially available porcine kits from Biotrend Chemikalien GmbH (Köln, Germany). The concentration of plasma leptin was analyzed by a double antibody enzyme immunoassay as described by Sauerwein et al. [16]. The minimal detectable concentration in the assay was 0.3 ng/ml, and the intra- und interassay coefficients of variance (CV) were 3.6% and 7.8%. Plasma IGF-I concentrations were determined using a competitive electrochemiluminescence immunoassay as described by Rehfeldt et al. [17]. The assay was modified by the use of receptor grade recombinant hIGF-I (GPB, Mediagnost, Reutlingen, Germany) as a standard and 25 µl as sample volume based on the extraction of a 50 µl plasma sample according to the DSL (Sinsheim, Germany) extraction protocol for IGF-I. Intra- and inter-assay CV were 3.5% and 7.6%, respectively. The P4 concentration was determined by a direct ^3^H-RIA in 10 µl duplicates as previously described [18]. The test sensitivity was 7 pg/ml, and intra- and inter-assay CV were 7.6% and 9.8%. Plasma total cortisol was determined as previously described by Kanitz et al. [19]. The test sensitivity was 8.1 nmol/l, and intra- and inter-assay CV were 8.2% and 9.8%, respectively.

The glucagon∶insulin ratio was calculated as the glucagon concentration in nmol/l divided by the insulin concentration in nmol/l. The insulin∶glucose ratio was calculated as the insulin concentration in pmol/l divided by the glucose concentration in mmol/l.

### Calculations and statistical evaluation

In EXP1, one replicate had only AP and HP sows and was excluded from the analysis. Thus, plasma data of 16, 17, and 15 sows in the LP, AP, and HP group, respectively, were evaluated, if not given otherwise. In EXP2, plasma metabolite concentrations of 27 sows (AP, 9; HP, 9; LP, 9) were analyzed. Data was evaluated using SAS/STAT 9.2 (SAS Institute Inc., Cary, NC). Repeated measure ANOVA (PROC MIXED) was used to evaluate the effects of diet on sow plasma metabolite and hormone concentrations. The model included the fixed factors diet, replicate, the repeated factor time during pregnancy, and the interactions diet×replicate, and diet×time. Post hoc comparisons between different diet groups were made using the Tukey-Kramer test. The CORR procedure was used to calculate Pearson correlation coefficients within diets and pregnancy days between plasma metabolite and hormone concentrations and sow BW, sow BF as well as pregnancy outcome (total litter weight at birth, LW). The significance level was set at *P*<0.05. Results are reported as least square means ± SE.

## Results

### Postabsorptive plasma metabolites

Evaluation of blood metabolites revealed a diet effect for LDLC, HDLC, %HDLC, as well as serum urea and protein concentrations (**Table 2** **and** **3**). Plasma HDLC concentrations during pregnancy were reduced in the HP group compared with the AP group (*P*<0.01). In LP sows, LDLC concentrations were lowest compared with AP (*P*<0.01) and HP sows (*P*<0.01) (**Table 2**). Plasma urea concentrations were increased in HP (*P*<0.001) and decreased in LP sows (*P*<0.01). In addition, serum protein concentration was lower in LP compared with AP (*P*<0.01) and HP sows (*P*<0.05) (**Table 3**). The factor time during pregnancy was significant for plasma glucose, NEFA, TG, C, HDLC, %HDLC, urea, and serum protein concentrations. Irrespective of diets, plasma NEFA and TG increased in late pregnancy (**Table 3**). Diet×replicate interaction was apparent for the variable urea (*P*<0.01). The diet effect within replicates differed in magnitude but not in direction of the effect. With the exception of plasma glucose and NEFA concentrations, diet×time interactions were or tended to be significant for all other variables (**Table 2** **and** **3**). At 24 dpc, HDLC concentration was lower in HP than in AP sows (*P*<0.05). In addition, plasma %HDLC was lowest in HP sows at 24 and 66 dpc, and higher before than during pregnancy irrespective of the diet (*P*<0.05) (**Table 2**). Urea levels decreased in response to pregnancy in LP sows by 60 to 70% but increased in pregnant HP sows by 20 to 40% (*P*<0.05). Serum protein concentration decreased from mid to late pregnancy in LP and AP sows, and was lower in LP compared with AP and HP sows in late pregnancy (*P*<0.001) (**Table 3**).

**Table 2:** Basal plasma cholesterol concentrations of sows at 5 days before and 24, 66 and 108 days after insemination.

		Diet^1^						*P* value^2^				
	T	LP	SE	AP	SE	HP	SE	D	R	T	D×R	D×T
No. of animals		16		17		15						
Total cholesterol, mmol/l												
	−5	2.23^A^	0.11	2.64^A^	0.11	2.48^A^	0.12	0.091	<0.001	<0.001	0.360	0.066
	24	1.77^B^	0.10	2.14^B^	0.11	1.93^B^	0.11					
	66	1.94^A^	0.11	1.92^B^	0.11	2.20^A^	0.11					
	108	1.81^B^	0.11	1.81^B^	0.11	1.81^B^	0.11					
LDL cholesterol, mmol/l												
	−5	0.79	0.09	1.03	0.10	0.82	0.10	0.002	0.261	0.343	0.917	0.060
	24	0.50^b^	0.09	0.96^a^	0.09	0.88^ab^	0.09					
	66	0.68	0.09	0.71	0.09	0.97	0.09					
	108	0.59	0.09	0.70	0.09	0.85	0.09					
HDL cholesterol, mmol/l												
	−5	1.32^A^	0.06	1.51^A^	0.06	1.42^A^	0.06	0.015	<0.001	<0.001	0.354	0.071
	24	0.95^ab^^,^ B	0.06	1.06^a^^,^ B	0.06	0.77^b^^,^ B	0.06					
	66	0.99^B^	0.06	0.96^B^	0.06	0.83^B^	0.06					
	108	0.96^B^	0.06	0.87^B^	0.06	0.78^B^	0.06					
HDL cholesterol, %												
	−5	60.5^A^	2.8	57.8^A^	2.8	60.0^A^	3.0	<0.001	0.001	<0.001	0.085	0.035
	24	54.4^a^^,^ B	2.5	48.2a,b ^,^ B	2.5	39.9^b^^,^ B	2.7					
	66	50.2^a^^,^ B	2.5	52.2^a^^,^ B	2.5	37.2^b^^,^ B	2.7					
	108	54.4^B^	2.6	50.9^a^^,^ B	2.5	42.5^B^	2.7					

Sows were fed isoenergetic diets with low protein∶high carbohydrate (6.5% crude protein, LP), adequate protein∶carbohydrate (12.1% crude protein, AP), and high protein∶low carbohydrate (30% crude protein, HP) ratios throughout pregnancy (EXP1).

a–c Within a row, values not sharing a common superscript differ significantly (*P*<0.05) according to Tukey post hoc test.

A–C Within diet and variable, values not sharing a common superscript differ significantly (*P*<0.05) according to Tukey post hoc test.

1 Values are least squares means ± SE per diet over 7 replicates.

2 ANOVA PROC MIXED, D = diet, R = replicate, T = time in days after insemination. D×R = interaction of diet×replicate. D×T = interaction of diet×time.

**Table 3:** Basal plasma metabolite concentrations of sows at 5 days before and 24, 66 and 108 days after insemination.

		Diet^1^						*P* value^2^				
	T	LP	SE	AP	SE	HP	SE	D	R	T	D×R	D×T
No. of animals		16		17		15						
Glucose, mmol/l												
	−5	4.24	0.13	4.14	0.12	4.02	0.14	0.407	0.036	0.001	0.316	0.898
	24	3.95	0.12	3.99	0.12	3.94	0.13					
	66	3.87	0.12	3.65	0.12	3.76	0.13					
	108	4.24	0.12	4.03	0.12	4.20	0.13					
NEFA, mmol/l												
	−5	0.41	0.21	0.43	0.20	0.56	0.22	0.623	0.018	<0.001	0.129	0.917
	24	0.54	0.19	0.92	0.18	0.76	0.20					
	66	0.89	0.19	1.00	0.18	0.99	0.20					
	108	1.63	0.20	1.73	0.18	1.74	0.20					
Triacylglycerol, mmol/l												
	−5	0.29^A^	0.04	0.26^A^	0.04	0.22^A^	0.04	0.910	0.402	<0.001	0.775	0.013
	24	0.35^A^	0.05	0.33^A^	0.04	0.35^B^	0.04					
	66	0.40^B^	0.04	0.35^B^	0.04	0.34^B^	0.04					
	108	0.57^B^	0.04	0.64^B^	0.04	0.72^C^	0.04					
Urea, mmol/l												
	−5	4.6^A^	0.2	4.3^A^	0.2	4.9^A^	0.3	<0.001	0.011	<0.001	0.006	<0.001
	24	1.4^c^^,^ B	0.2	2.6^b^^,^ B	0.2	6.9^a^^,^ B	0.2					
	66	1.5^c^^,^ B	0.2	2.5^b^^,^ B	0.2	6.3^a^^,^ B	0.2					
	108	1.7^c^^,^ B	0.2	3.0^b^^,^ B	0.2	7.1^a^^,^ B	0.2					
Serum protein, mg/ml												
	−5	77.4^A^	1.3	78.4	1.2	78.6	1.4	0.002	<0.001	<0.001	0.607	0.002
	24	73.5^A^	1.2	76.7	1.1	75.2	1.3					
	66	75.6^A^	1.2	80.2^A^	1.1	78.3	1.3					
	108	67.6^b^^,^ B	1.2	75.1^a^^,^ B	1.1	77.2^a^	1.3					

Sows were fed isoenergetic diets with low protein∶high carbohydrate (6.5% crude protein, LP), adequate protein∶carbohydrate (12.1% crude protein, AP), and high protein∶low carbohydrate (30% crude protein, HP) ratios throughout pregnancy (EXP1).

a–c Within a row, values not sharing a common superscript differ significantly (*P*<0.05) according to Tukey post hoc test.

A–C Within diet and variable, values not sharing a common superscript differ significantly (*P*<0.05) according to Tukey post hoc test.

1 Values are least squares means ± SE per diet over 7 replicates.

2 ANOVA PROC MIXED, D = diet, R = replicate, T = time in days after insemination. D×R = interaction of diet×replicate. D×T = interaction of diet×time.

### Postabsorptive plasma hormones

Glucagon, IGF-I, P4, and cortisol concentrations were affected by diet and time (**Table 4** **and** **5**). Tukey test indicated that glucagon levels in HP sows were higher than in AP (*P*<0.05) whereas IGF-I concentrations were lower in LP than in AP sows at 24 and 66 dpc (*P*<0.05). Concentrations of P4 were higher in HP than in LP (*P*<0.05) with intermediate values in AP sows. Cortisol levels were lower in HP compared with AP sows (*P*<0.05). In late pregnancy, cortisol concentrations were increased only in LP and AP sows compared with early and mid pregnancy (**Table 5**). The significant diet×replicate interaction for the variables IGF-I and leptin revealed that diet effect in replicates differed in magnitude but not in direction of the effect.

**Table 4:** Basal plasma insulin and glucagon concentrations as well as insulin ratios of sows at 5 days before and 24, 66 and 108 days after insemination.

		Diet^1^						*P* value^2^				
	T	LP	SE	AP	SE	HP	SE	D	R	T	D×R	D×T
No. of animals		16		17		15						
Insulin, µU/ml												
	−5	10.3	1.6	9.5	1.7	9.4	1.9	0.753	<0.001	0.109	0.123	0.441
	24	7.1	1.5	9.4	1.6	9.8	1.6					
	66	4.4	1.5	8.6	1.6	6.5	1.6					
	108	9.2	1.5	7.9	1.6	7.9	1.6					
Glucagon, pg/ml												
	−5	104.8	6.2	97.7	6.5	103.3	6.8	0.042	0.051	<0.001	0.504	0.265
	24	69.6	5.7	64.0	6.0	83.8	6.2					
	66	60.2	5.7	69.5	5.9	73.3	6.4					
	108	70.1	5.7	66.9	6.4	83.8	6.5					
Ratios												
Glucagon to Insulin, nmol/nmol				(13)		(13)						
	−5	0.6	0.6	2.1	0.6	0.5	0.6	0.177	0.380	0.734	0.219	0.225
	24	0.5	0.5	2.3	0.5	0.4	0.5					
	66	0.6	0.5	0.9	0.6	0.5	0.6					
	108	0.8	0.5	0.3	0.6	1.2	0.6					
Insulin to Glucose, pmol/mmol		(15)		(14)		(13)						
	−5	17.3	2.3	14.4	2.3	17.2	2.6	0.251	0.005	0.094	0.246	0.213
	24	12.3	2.0	10.9	2.2	17.3	2.1					
	66	8.2	2.0	14.4	2.3	11.9	2.3					
	108	13.3	2.2	13.3	2.3	13.2	2.2					

Sows were fed isoenergetic diets with low protein∶high carbohydrate (6.5% crude protein, LP), adequate protein∶carbohydrate (12.1% crude protein, AP), and high protein∶low carbohydrate (30% crude protein, HP) ratios throughout pregnancy (EXP1).

1 Values are least squares means ± SE per diet over 7 replicates. Values in parenthesis indicate numbers of sows within dietary group measured for certain plasma parameters if different from the number of sows generally used per group.

2 ANOVA PROC MIXED, D = diet, R = replicate, T = time in days after insemination. D×R = interaction of diet×replicate. D×T = interaction of diet×time.

**Table 5:** Basal plasma IGF-I, progesterone, leptin and cortisol concentrations of sows at 5 days before and 24, 66 and 108 days after insemination.

		Diet^1^						*P* value^2^				
	T	LP	SE	AP	SE	HP	SE	D	R	T	D×R	D×T
No. of animals		16		17		15						
IGF-I, ng/ml												
	−5	294.3^A^	9.4	303.8^A^	8.8	286.6^A^	9.3	0.041	<0.001	<0.001	0.030	<0.001
	24	210.6^b^^,^ B	9.3	256.5^a^^,^ B	8.8	239.7^ab^^,^ B	9.3					
	66	150.5^b^^,^ C	9.4	202.1^a^^,^ C	8.8	176.0^ab^^,^ C	9.3					
	108	182.4^C^	10.1	161.1^C^	9.4	158.1^C^	9.8					
Progesterone, mg/ml		(14)		(13)		(12)						
	−5	0.2	0.6	0.3	0.6	0.4	0.6	0.021	0.026	<0.001	0.247	0.118
	24	13.1^b^	0.6	14.0^ab^	0.6	16.5^a^	0.6					
	66	12.4	0.6	13.2	0.6	14.6	0.6					
	108	9.7	0.6	9.7	0.6	9.5	0.6					
Leptin, ng/ml		(14)		(16)								
	−5	6.0	0.6	5.5	0.6	5.9	0.6	0.219	0.007	0.076	0.043	0.874
	24	6.2	0.5	5.4	0.5	5.4	0.6					
	66	6.5	0.5	5.2	0.6	5.2	0.6					
	108	6.3	0.5	5.0	0.6	4.9	0.6					
Cortisol, nmol/l												
	−5	47.4^A^	6.8	60.5^A^	6.6	52.2	7.5	0.025	<0.001	<0.001	0.254	0.294
	24	63.1^A^	6.5	66.8A,B	6.4	51.9	7.1					
	66	66.0^A^	6.4	86.2A,B	6.4	60.4	7.3					
	108	94.7^B^	6.4	90.9^B^	6.4	73.8	7.3					

Sows were fed isoenergetic diets with low protein∶high carbohydrate (6.5% crude protein, LP), adequate protein∶carbohydrate (12.1% crude protein, AP), and high protein∶low carbohydrate (30% crude protein, HP) ratios throughout pregnancy (EXP1).

a–c Within a row, values not sharing a common superscript differ significantly (*P*<0.05) according to Tukey post hoc test.

A–C Within diet and variable, values not sharing a common superscript differ significantly (*P*<0.05) according to Tukey post hoc test.

1 Values are least squares means ± SE per diet over 7 replicates. Values in parenthesis indicate numbers of sows within dietary group measured for certain plasma parameters if different from the number of sows generally used per group.

2 ANOVA PROC MIXED, D = diet, R = replicate, T = time in days after insemination. D×R = interaction of diet×replicate. D×T = interaction of diet×time.

### Diurnal plasma metabolites in late gestation

**Figure 1:**
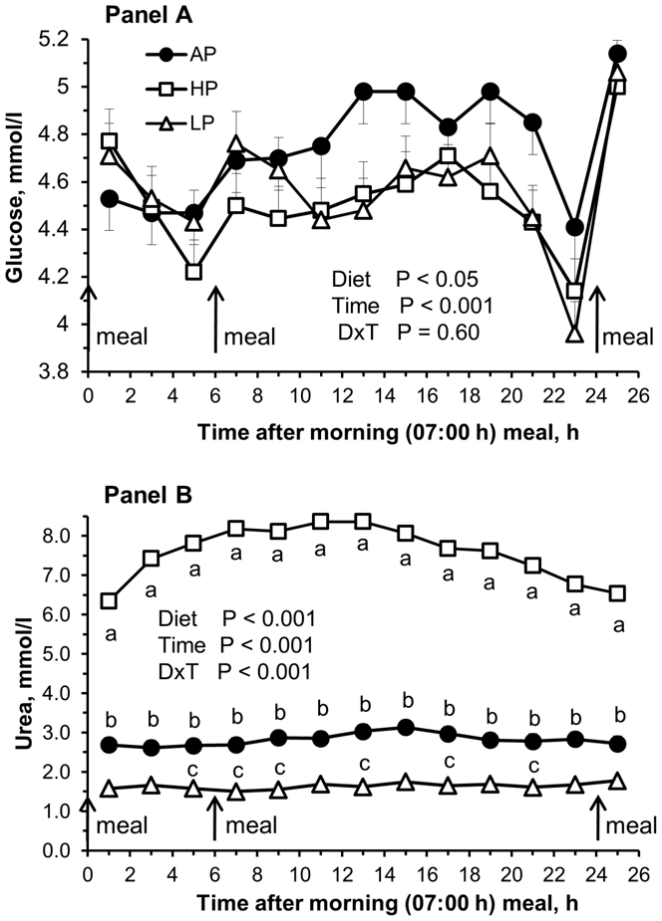
Diurnal plasma glucose and urea concentrations at 92 dpc. Diurnal plasma metabolite concentrations of sows at 92 dpc fed isoenergetic diets with low protein∶high carbohydrate (LP; open triangles), adequate protein∶carbohydrate (AP; closed circles), and high protein∶low carbohydrate (HP; open squares) ratios throughout pregnancy. Values are depicted from one hour after feeding the morning meal (50% of the daily allowance at 07:00h) to the midday meal (13:00 h) until the next day one hour after morning meal (08:00 h). Panel A, glucose; panel B, urea; Values are least square means ± SE, n = 9 per group. Inserts depict *P* values for the main factors diet and time and interactions. ^a,b,c^ Within time points, values with different lower case letters indicate significant differences (*P*<0.05) between diet groups.

**Figure 2:**
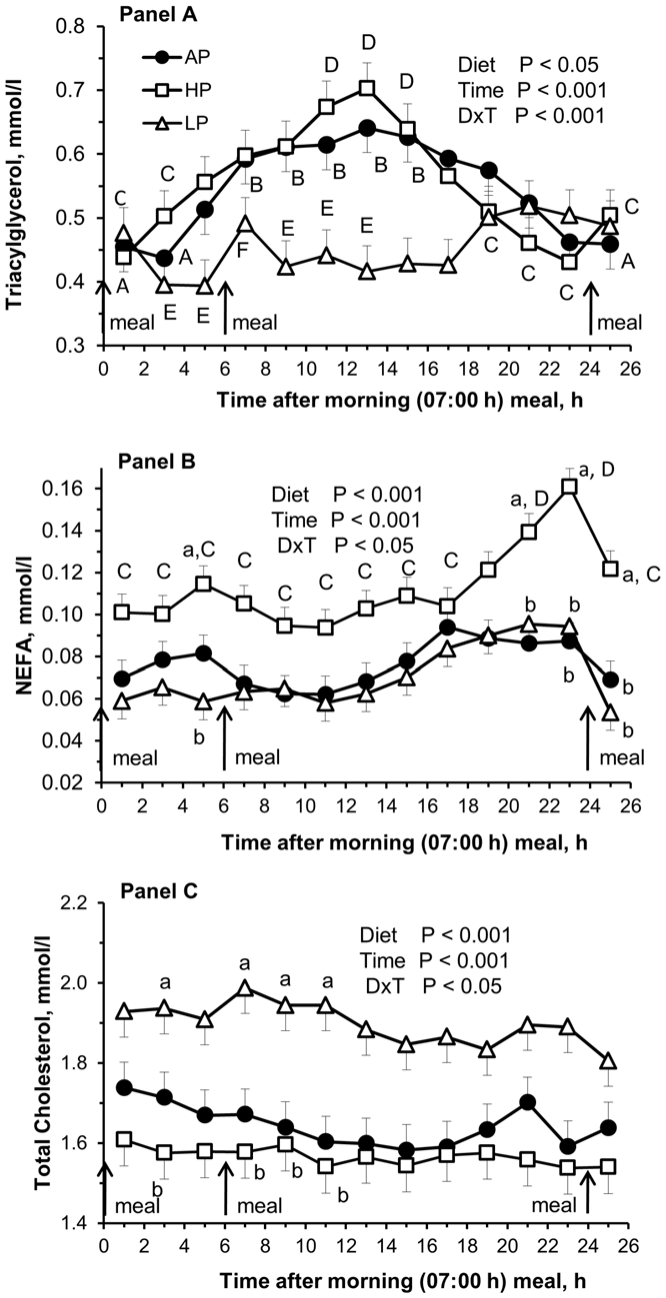
Diurnal plasma triacylglycerol, non-esterified fatty acid and total cholesterol concentrations at 92 dpc. Diurnal plasma metabolite concentrations of sows at 92 dpc fed isoenergetic diets with low protein∶high carbohydrate (LP; open triangles), adequate protein∶carbohydrate (AP; closed circles), and high protein∶low carbohydrate (HP; open squares) ratios throughout pregnancy. Values are depicted from one hour after feeding the morning meal (50% of the daily allowance at 07:00h) to the midday meal (13:00 h) until the next day one hour after morning meal (08:00 h). Panel A, triacylglycerol; panel B, non-esterified fatty acids (NEFA); panel C, total cholesterol. Values are least square means ± SE, n = 9 per group. Inserts depict *P* values for the main factors diet and time and interactions. ^a,b,c^ Within time points, values with different lower case letters indicate significant differences (*P*<0.05) between diet groups. ^A,B^ Within diet AP, values with different upper case letters indicate differences (*P*<0.05) between time points. ^C,D^ Within diet HP, values with different upper case letters indicate differences (*P*<0.05) between time points. ^E,F^ Within diet LP, values with different upper case letters indicate differences (*P*<0.05) between time points.

Pregnancy diet affected plasma values of glucose, urea (**Figure 1, *A–B***), TG, NEFA and C (**Figure 2, *A–C***). Post-hoc tests indicated that glucose concentrations were lower in HP sows (*P*<0.05) as compared with AP sows (**Figure 1** ***A***). In HP sows, urea concentrations reached approximately 300% of the values observed in AP sows (*P*<0.001), whereas in LP sows urea levels were approximately at 60% of the control values (*P*<0.001) (**Figure 1** ***B***). Plasma TG levels tended to be less in LP than in AP and HP sows (*P*≤0.1) (**Figure 2** ***A***). Seven hours after the midday (13:00 h) meal, TG values reached a nadir in AP and HP sows, but returned to baseline values prior to the next meal in the morning. Plasma NEFA concentrations were higher in HP as compared with AP (*P*<0.01) and LP sows (*P*<0.001) (**Figure 2** ***B***). In the HP group, NEFA concentration was highest one hour before the morning meal and decreased thereafter. Plasma C concentrations were higher in LP than in AP (*P*<0.01) and HP sows (*P*<0.001) (**Figure 2** ***C***).

### Pearson correlation coefficients

Correlations of postabsorptive metabolite and hormone concentrations with total LW at birth, sow BW, and BF within diet in early (24 dpc), mid (66 dpc), or late (108 dpc) pregnancy are shown in **Table 6**.

**Table 6:** Significant Pearson correlation coefficients within diets and day after insemination between plasma metabolite and hormone concentrations and sow BW, sow BF as well as pregnancy outcome (total litter weight at birth, LW).

	Diet																
	LP						AP					HP					
dpc	BW		BF		LW		BW		BF	LW		BW		BF		LW	
24	P4	−0.86	SP	−0.61	C	−0.51	NEFA	−0.51	-	-		-		NEFA	−0.58	-	
	-		LDLC	−0.55	-		-		-	-		-		-		-	
	-		I	+0.50	-		-		-	-		-		-		-	
	-		I/G	+0.55	-		-		-	-		-		-		-	
66	-		-		-		SP	−0.50	-	I	+0.68	-		CS	−0.56	NEFA	+0.63
	-		-		-		-		-	I/G	+0.65	-		-		-	
	-		-		-		-		-	Gg/I	−0.61	-		-		-	
108	-		-		-		G	+0.47	-	-		IGF	+0.52	I/G	+0.52	NEFA	+0.51
	-		-		-		-		-	-		P4	−0.61	-		-	

Variables: dpc, days post coitum; BW, body weight; BF, back fat thickness; LW, total litter weight at birth.

Concentrations: C, total cholesterol; CS, cortisol; G, glucose; Gg/I, glucagon to insulin ratio; I, insulin; I/G, insulin to glucose ratio; IGF, insulin like growth factor-I; LDLC, low density lipoprotein cholesterol; NEFA, non-esterified fatty acid; P4, progesterone; SP, serum protein.

### Correlations with sow BW

In regard to BW, in AP sows at 24 dpc there was a negative correlation with NEFA (**Table 6**). In LP sows, BW correlated negatively with P4. In mid pregnancy, AP sows showed a negative correlation for BW with serum protein. In late pregnancy, in AP sows a positive correlation was observed between BW and glucose, and between BW and IGF-I in HP sows, whereas correlation between BW and P4 was negative in the latter group (**Table 6**).

### Correlations with sow BF

In LP sows, negative correlations were detected at 24 dpc between BF and serum protein, and BF and LDLC, whereas positive correlations were observed between BF and insulin and insulin∶glucose ratio. HP sows showed a negative relationship between BF and NEFA (**Table 6**). At 66 dpc, in HP sows correlation between BF and cortisol was negative. In late gestation, BF showed a positive correlation with insulin∶glucose ratio in HP sows (**Table 6**).

### Correlations with total LW at birth

No correlations at all were observed between sow BW and LW. There was a significant negative relationship between LW and C at 24 dpc in LP sows (**Table 6**). In mid pregnancy, we found in the control group AP positive correlations of LW with insulin and insulin/glucose ratio, and a negative correlation with the glucagon/insulin ratio (**Table 6**). In HP sows, the correlation of LW with NEFA was positive at 66 and 108 dpc. No correlation of LW with any other variable was found in LP sows at 66 and 108 dpc.

## Discussion

There is insufficient data available on the long-term effect of high protein diets, especially in reproductive females. We observed IUGR in offspring from sows fed a diet with high protein∶carbohydrate ratio, similar to that found in sows fed a low protein diet throughout pregnancy [4]. To identify the specific diet-related metabolic constraints in HP and LP sows which hampered normal pregnancy BW gain, adipose tissue development and fetal mass accretion, we investigated maternal plasma metabolite and hormone concentrations during the course of pregnancy and calculated Pearson correlations as a reflection of the maternal-fetal interaction.

Our results indicate that IUGR in the HP group was associated with a low energy status as suggested by 1.8 times the diurnal plasma NEFA concentrations of control sows. This corresponds to a NEFA increase of a similar magnitude as observed after 24 h fasting in pigs [20]. Progressing pregnancy was associated with an increase in lipolysis and fat oxidation as shown previously in humans [21], which is in line with the increasing NEFA concentrations in pregnant sows of this study. The negative correlation of BF with plasma cortisol in HP sows also suggests increased lipolysis under the action of glucocorticoids in mid pregnancy. We found a positive relationship between NEFA and LW in mid and late pregnancy of HP sows suggesting that sows mobilizing body reserves to a larger extent under this catabolic condition were better able to support fetal body mass at the onset of mass accretion. This, however, was not the case in the two other dietary groups. On the contrary, under normal dietary conditions (control group) LW was positively affected by insulin and negatively by glucagon in line with a normal pregnancy anabolism.

The higher basal plasma glucagon and lower diurnal glucose levels suggest a maternal glucose deficit in HP sows. This interpretation is supported by the low body fat content and compromised body mass gain in this pig model [4], which is a consequence of a lack of carbohydrates to be used for de novo lipogenesis as reported recently in rats fed a high protein diet [22]. Effects of high protein diets on body fat and metabolic characteristics have been earlier shown in humans [10], [23] and non-pregnant and pregnant animals [22], [24], [25]. In rats, prandial plasma glucose and insulin were reported to be lower when a high protein diet was fed [26]. In late pregnancy, HP sows but not LP and AP sows presented a positive relationship between BF and insulin∶glucose ratio indicating the involvement of insulin for the deposition of body energy reserves also in this treatment group.

Adaptive responses in nitrogen metabolism such as decreased plasma urea concentration were apparent early in pregnancy of AP and LP sows. This effect is also known in humans and reflects a reduced urea synthesis associated with protein accretion by the mother [27]. In contrast and not surprising, the high dietary protein intake resulted in an increase of serum urea concentrations in the HP sows, distinctly different from the two other groups. It has been shown in rodent and sheep models that increased concentration of ammonium and high periconceptional protein intake, respectively, decreased the number of developing blastocysts, perturbs embryonic metabolism and leads to impaired fetal growth [28]–[30].

Diet-dependent differences in postabsorptive plasma concentrations occurred in relation to lipoprotein and C metabolism. Lowest HDLC values were observed in HP sows whereas LDLC concentrations were lowest in LP sows as compared with control sows. This suggests divergent alterations in the lipoprotein metabolism of LP and HP sows. Since the experimental diets were comparably low in fat and free of C [4], it is assumed that these differences are related to the different dietary protein∶carbohydrate ratio. Lower levels of HDLC and %HDLC in HP sows may suggest an increased HDL clearance because of lower peripheral levels of C, or could indicate a reduced synthesis of Apo-A1, the main apolipoprotein of HDL particles as observed earlier under conditions of low glucose and high NEFA concentrations [31]. Relative high body fatness of LP sows suggests a necessity for more TG and C trafficking between liver and periphery which might have relatively increased the need for apolipoproteins. In addition, due to the deficit of indispensable AA, as indicated by the lower serum protein values in this group, apolipoprotein synthesis might have been compromised [32], [33]. The lower LDLC values are likely related to a lower VLDL synthesis. In addition, in the LP group, higher diurnal concentrations of C and postabsorptive %HDLC suggest a higher C synthesis and/or a lower C excretion via bile.

In fetal development, C is important as an essential structural component of cell membranes and thus fetal growth. Maternal plasma lipoprotein C could play a nutritive role in embryonic and/or fetal development. It has been shown that a low maternal HDLC concentration was related to a 15% reduced fetal mass, and sterol homeostasis in fetal tissues was affected by maternal plasma C concentration [34], [35]. We found negative correlations between LW and C in LP sows. This suggests that there might be some relationship between C metabolism and IUGR in these pregnant sows which deserves further investigation.

Plasma P4 is produced by the corpus luteum graviditatis and is responsible for implantation, early embryo development and maintenance of pregnancy. In agreement with earlier findings in sows, P4 increased in the maternal plasma during the first four weeks of pregnancy and decreased slowly thereafter [36]. The change in P4 is reflected in the dip of plasma C concentrations from pre-pregnancy to 24 dpc because P4 is synthesized from C. In swine, LDL is the main source of C for steroidogenesis [37]. In LP sows, P4 concentration was lower in early pregnancy than in HP sows, and BW was inversely correlated with P4 in this group. This might be related to a decreased luteal production of P4 possibly due to changes in C transport to the corpus luteum associated with reduced LDLC concentration. In pregnant rats fed low protein diet, plasma P4 concentrations were also lower in mid pregnancy, and low plasma P4 concentrations in early pregnancy were associated with embryonic loss in sows [38], [39]. This might indicate the involvement of P4 in the lower growth of LP fetuses.

In spite of the difference in body fatness among the three groups of sows [4], we could not detect differences in plasma leptin concentration. This is surprising because circulating levels of leptin in humans and rodents correlate with body adiposity, and in rats and humans maternal leptin concentrations increased until mid to late pregnancy [21], [40]. In humans, placental production of leptin contributes to leptin levels in the maternal circulation which is in contrast to rats, where hyperleptinaemia during pregnancy is mainly derived from increasing body fat [40]. Thus, it appears that pregnant adolescent sows do not develop hyperleptinemia as described for humans and rodents.

The HP diet caused lower total cortisol concentrations in plasma compared with control sows, an effect which was also observed in fasting rats after feeding a high protein∶low carbohydrate diet over a period of 6 months [41]. The increased uptake of tryptophan with the HP diet may be one explanation for this difference. It was shown previously that tryptophan supplementation in pigs and humans can lead to reduced basal and stress-induced plasma cortisol concentrations, possibly caused by increased brain serotonin activity and increased negative feedback on the hypothalamic-pituitary-adrenal axis [42]–[46]. Alterations in cortisol levels may also be caused by the different protein∶carbohydrate ratios in the diets. In a human study, it was shown that the cortisol response to consumption of protein was lower than the response to carbohydrate [47]. In addition, it can be speculated that in HP sows cortisol biosynthesis may be disturbed either by lower C availability or by altered enzyme activities as shown in hepatocytes of rats fed a high protein diet [48]. Thus, the negative association between cortisol and BF in mid pregnancy of HP sows can be interpreted as a reflection of the alteration in C and lipoprotein metabolism under conditions of a high protein diet.

The IGF system is considered as one of the most important regulators of fetal growth but the extent to which IGFs influence the mother and/or placenta are dependent on the species and maternal factors, including nutrition [49]. In humans and other species, plasma IGF-I levels increase during pregnancy [49]. In contrast, we found that plasma IGF-I concentrations decreased during pregnancy irrespective of the diet which confirms previous results in pigs [50], [51]. In LP sows, IGF-I concentrations were lower than in controls as previously shown in protein-deficient rats [52]. In pregnant sows and adolescent ewes with high dietary energy intake, a higher plasma IGF-I level was found, indicating that dietary energy but not high protein intake leads to increased IGF-I values [53]–[55]. In fetuses of rat dams fed a low protein diet, plasma and liver IGF-I were lower than in controls [56]. Maternal plasma IGFs correlate positively with fetal growth and birth weight in several species [50]. The positive relationship between BW and IGF-I in late pregnancy in the HP sows suffering from metabolic energy deficit might mean that maternal nutrients are repartitioned in favor of maternal anabolism.

Overfeeding during adolescent pregnancy in ewes has been shown to be associated with an adverse outcome for the offspring [2], [3]. We demonstrated that feeding of a high protein∶low carbohydrate diet in pregnant adolescent sows leads to IUGR associated with massively reduced body fat in the pregnant sow, which contrasts with the situation of a protein deficiency and high carbohydrate intake [4]. Taken together, diet-dependent changes in maternal body weight, body fat, or plasma metabolites and hormones relate to IUGR and provide clues on the underlying metabolic reasons. Thus, a high protein∶low carbohydrate diet in pregnant sows stimulates lipolysis, ureagenesis and gluconeogenesis as reflected by increased NEFA and urea and lower glucose concentrations. This might cause a metabolic energy and net glucose deficit that partly explains the reduced maternal body fat stores and diet-dependent IUGR observed in this adolescent porcine model. In contrast, in sows fed a low protein∶high carbohydrate diet, absolute deficiency of indispensable AA was possibly the main determinant of IUGR, as indicated by decreased urea and serum protein levels, while a possible effect of maternal steroidogenesis and fetal cholesterol availability cannot be excluded considering the altered plasma lipoprotein pattern. In further studies it would be important to investigate whether this negative effect of a high protein∶low carbohydrate diet on fetal growth is specific for adolescent pregnancies which are characterized by a superimposition of increased nutritional requirements for the maintenance of maternal together with fetal growth.

## References

[1] Scholl TO, Hediger ML, Schall JI, Khoo CS, Fischer RL (1994). Maternal growth during pregnancy and the competition for nutrients.. Am J Clin Nutr.

[2] Wallace JM, Milne JS, Aitken RP (2010). Effect of weight and adiposity at conception and wide variations in gestational dietary intake on pregnancy outcome and early postnatal performance in young adolescent sheep.. Biol Reprod.

[3] Wallace JM, Luther JS, Milne JS, Aitken RP, Redmer DA (2006). Nutritional modulation of adolescent pregnancy outcome – a review.. Placenta.

[4] Rehfeldt C, Lang IS, Görs S, Hennig U, Kalbe C (2011). Limited and excess dietary protein during gestation affects growth and compositional traits in gilts and impairs offspring fetal growth.. J Anim Sci.

[5] Rehfeldt C, Lefaucheur L, Block J, Stabenow B, Brüssow KP (2011). Limited and excess protein intake of pregnant gilts differently affects body composition and cellularity of skeletal muscle and subcutaneous adipose tissue of newborn and weanling piglets.. Eur J Nutr.

[6] Spurlock ME, Gabler NK (2008). The development of porcine models of obesity and the metabolic syndrome.. J Nutr.

[7] Langley-Evans SC (2001). Fetal programming of cardiovascular function through exposure to maternal undernutrition.. Proc Nutr Soc.

[8] Christian P (2009). Prenatal origins of undernutrition.. Nestle Nutr Workshop Ser Pediatr Program.

[9] Halton TL, Hu FB (2004). The effects of high protein diets on thermogenesis, satiety and weight loss: A critical review.. J Am Coll Nutr.

[10] Nordmann AJ, Nordmann A, Briel M, Keller U, Yancy WS (2006). Effects of low-carbohydrate vs low-fat diets on weight loss and cardiovascular risk factors: a meta-analysis of randomized controlled trials.. Arch Intern Med.

[11] Peters JC, Mahan DC (2008). Effects of dietary organic and inorganic trace mineral levels on sow reproductive performances and daily mineral intakes over six parities.. J Anim Sci.

[12] GfE (Gesellschaft für Ernährungsphysiologie) (2006). Empfehlungen zur Energie- und Nährstoffversorgung von Schweinen (Recommendations of energy and nutrient intake in pigs)..

[13] Wiseman TG, Mahan DC, Moeller SJ, Peters JC, Fastinger ND (2007). Phenotypic measurements and various indices of lean and fat tissue development in barrows and gilts of two genetic lines from twenty to one hundred twenty-five kilograms of body weight.. J Anim Sci.

[14] McPherson RL, Ji F, Wu G, Blanton JR, Kim SW (2004). Growth and compositional changes of fetal tissues in pigs.. J Anim Sci.

[15] Rodriguez H, Kunavongkrit A (1983). Chronical venous catheterization for frequent blood sampling in unrestrained pigs.. Act Vet Scand.

[16] Sauerwein H, Heintges U, Hennies M, Selhorst T, Daxenberger A (2004). Growth hormone induced alterations of leptin serum concentrations in dairy cows as measured by a novel enzyme immuno assay.. Livest Prod Sci.

[17] Rehfeldt C, Kuhn G, Nürnberg G, Kanitz E, Schneider F (2001). Effects of exogenous somatotropin during early gestation on maternal performance, fetal growth, and compositional traits in pigs.. J Anim Sci.

[18] Schneider F, Brüssow K-P (2006). Effects of a preovulatory administered depot gonadotrophin-releasing hormone agonist on reproductive hormone levels and pregnancy outcome in gilts.. Reprod Fert Dev.

[19] Kanitz E, Puppe B, Tuchscherer M, Heberer M, Viergutz T (2009). A single exposure to social isolation in domestic piglets activates behavioural arousal, neuroendocrine stress hormones, and stress-related gene expression in the brain.. Physiol Behav.

[20] Toscano MJ, Lay DC, Craig BA, Pajor EA (2007). Assessing the adaptation of swine to fifty-seven hours of feed deprivation in terms of behavioral and physiological responses.. J Anim Sci.

[21] Okereke NC, Huston-Presley L, Amini SB, Kalhan S, Catalano PM (2004). Longitudinal changes in energy expenditure and body composition in obese women with normal and impaired glucose tolerance.. Am J Physiol Endocrinol Metab.

[22] Stepien M, Gaudichon C, Azzout-Marniche D, Fromentin G, Tomé D (2010). Postprandial nutrient partitioning but not energy expenditure is modified in growing rats during adaptation to a high-protein diet.. J Nutr.

[23] Noakes M, Keogh JB, Foster PR, Clifton PM (2005). Effect of an energy-restricted, high-protein, low-fat diet relative to a conventional high-carbohydrate, low-fat diet on weight loss, body composition, nutritional status, and markers of cardiovascular health in obese women.. Am J Clin Nutr.

[24] Kucia M, Langhammer M, Görs S, Albrecht E, Hammon HM (2011). High-protein diet during gestation and lactation affects mammary gland mRNA abundance, milk composition and pre-weaning litter growth in mice.. Animal.

[25] Jia Y, Hwang SY, House JD, Ogborn MR, Weiler HA (2010). Long-term high intake of whole proteins results in renal damage in pigs.. J Nutr.

[26] Stepien M, Gaudichon C, Fromentin G, Even P, Tomé D (2011). Increasing protein at the expense of carbohydrate in the diet down-regulates glucose utilization as glucose sparing effect in rats.. PLoS One.

[27] Kalhan SC, Devapatla S (1999). Pregnancy, insulin resistance and nitrogen accretion.. Curr Opin Clin Nutr Metab Care.

[28] Lane M, Gardner DK (2003). Ammonium induces aberrant blastocyst differentiation, metabolism, pH regulation, gene expression and subsequently alters fetal development in the mouse.. Biol Reprod.

[29] Gardner DK, Stilley KS, Lane M (2004). High protein diet inhibits inner cell mass formation and increases apoptosis in mouse blastocysts developed in vitro by increasing the levels of ammonium in the reproductive tract.. Reprod Fertil Dev.

[30] Meza-Herrera C, Ross T, Hallford D, Hawkins D, Gonzalez-Bulnes A (2010). High periconceptional protein intake modifies uterine and embryonic relationships increasing early pregnancy losses and embryo growth retardation in sheep.. Reprod Domest Anim.

[31] Mooradian AD, Haas MJ, Wong NC (2006). The effect of select nutrients on serum high-density lipoprotein cholesterol and apolipoprotein A-I levels.. Endocr Rev.

[32] Jahoor F, Bhattiprolu S, Del Rosario M, Burrin D, Wykes L (1996). Chronic protein deficiency differentially affects the kinetics of plasma proteins in young pigs.. J Nutr.

[33] Jackson AA, Phillips G, McClelland I, Jahoor F (2001). Synthesis of hepatic secretory proteins in normal adults consuming a diet marginally adequate in protein.. Am J Physiol Gastrointest Liver Physiol.

[34] McConihay JA, Honkomp AM, Granholm NA, Woollett LA (2000). Maternal high density lipoproteins affect fetal mass and extra-embryonic fetal tissue sterol metabolism in the mouse.. J Lipid Res.

[35] McConihay JA, Horn PS, Woollett LA (2001). Effect of maternal hypercholesterolemia on fetal sterol metabolism in the Golden Syrian hamster.. J Lipid Res.

[36] Robertson HA, King GJ (1974). Plasma concentrations of progesterone, oestrone, oestradiol-17ß and of oestrone sulphate in the pig at implantation, during pregnancy and at parturition.. J Reprod Fert.

[37] Grummer RR, Carroll DJ (1988). A review of lipoprotein cholesterol metabolism: Importance to ovarian function.. J Anim Sci.

[38] Fernandez-Twinn DS, Ozanne SE, Ekizoglou S, Doherty C, James L (2003). The maternal endocrine environment in the low-protein model of intra-uterine growth restriction.. Br J Nutr.

[39] Jindal R, Cosgrove JR, Foxcroft GR (1997). Progesterone mediates nutritionally induced effects on embryonic survival in gilts.. J Anim Sci.

[40] Henson MC, Castracane VD (2006). Leptin in pregnancy: an update.. Biol Reprod.

[41] Lacroix M, Gaudichon C, Martin A, Morens C, Mathé V (2004). A long-term high-protein diet markedly reduces adipose tissue without major side effects in Wistar male rats.. Am J Physiol Regul Integr Comp Physiol.

[42] Koopmans SJ, Ruis M, Dekker R, van Diepen H, Korte M (2005). Surplus tryptophan reduces plasma cortisol and noradrenaline concentrations and enhances recovery after social stress in pigs.. Physiol Behav.

[43] Koopmans SJ, Guzik AC, van der Meulen J, Dekker R, Kogut J (2006). Effects of supplemental L-tryptophan on serotonin, cortisol, intestinal integrity, and behavior in weanling piglets.. J Anim Sci.

[44] Guzik AC, Matthews JO, Kerr BJ, Bidner TD, Southern LL (2006). Dietary tryptophan effects on plasma an salivary cortisol and meat quality in pigs.. J Anim Sci.

[45] Markus CR, Olivier B, Panhuysen GE, Van Der Gugten J, Alles MS (2000). The bovine protein alpha-lactalbumin increases the plasma ratio of tryptophan to the other large neutral amino acids, and in vulnerable subjects raises brain serotonin activity, reduces cortisol concentration, and improves mood under stress.. Am J Clin Nutr.

[46] Firk C, Markus CR (2009). Mood and cortisol responses following tryptophane-rich hydrolyzed protein and acute stress in healthy subjects with high and low cognitive reactivity to depression.. Clin Nutr.

[47] Martens MJI, Rutters F, Lemmens SGT, Born JM, Westerterp-Plantenga MS (2010). Effects of single macronutrients on serum cortisol concentrations in normal weight men.. Physiol Behav.

[48] Rémésy C, Morand C, Demigné C, Farfournoux P (1988). Control of hepatic utilization of glutamine by transport processes or cellular metabolism in rats fed a high protein diet.. J Nutr.

[49] Sferruzzi-Perri AN, Owens JA, Pringle KG, Roberts CT (2011). The neglected role of insulin-like growth factors in the maternal circulation regulating fetal growth.. J Physiol.

[50] Govoni N, Parmeggiani A, Galeati G, Penazzi P, De Iasio R (2007). Acyl ghrelin and metabolic hormones in pregnant and lactating sows.. Reprod Domest Anim.

[51] Brown KR, Goodband RD, Tokach MD, Dritz SS, Nelssen JL (2007). Growth characteristics, blood metabolites, and insulin-like growth factor system components in maternal tissues of gilts fed L-carnitine through day seventy of gestation.. J Anim Sci.

[52] Divino Filho JC, Hazel SJ, Anderstam B, Bergström J, Lewitt M (1999). Effect of protein intake on plasma and erythrocyte free amino acids and serum IGF-I and IGFBP-I levels in rats.. Am J Physiol Endocrinol Metab.

[53] Musser RE, Davis DL, Dritz SS, Tokach MD, Nelssen JL (2004). Conceptus and maternal responses to increased feed intake during early gestation in pigs.. J Anim Sci.

[54] Nissen PM, Sorensen IL, Vestergaard M, Oksbjerg N (2005). Effects of sow nutrition on maternal and foetal serum growth factors and on foetal myogenesis.. Anim Sci.

[55] Wallace JM, Bourke D, Da Silva P, Aitken R (2001). Nutrient partitioning during adolescent pregnancy.. Reproduction.

[56] El-Khattabi I, Grégoire F, Remacle C, Reusens B (2003). Isocaloric maternal low-protein diet alters IGF-I, IGFBPs, and hepatocyte proliferation in the fetal rat.. Am J Physiol Endocrinol Metab.

